# Design considerations for engineering 3D models to study vascular pathologies *in vitro* ✩

**DOI:** 10.1016/j.actbio.2021.02.031

**Published:** 2021-02-27

**Authors:** Suzette T. Lust, Catherine M. Shanahan, Rebecca J. Shipley, Pablo Lamata, Eileen Gentleman

**Affiliations:** a Centre for Craniofacial and Regenerative Biology, King’s College London, London SE1 9RT, United Kingdom; b School of Biomedical Engineering and Imaging Sciences, King’s College London, London SE1 7EH, United Kingdom; c School of Cardiovascular Medicine and Sciences, King’s College London, London SE5 9NU, United Kingdom; d Institute of Healthcare Engineering and Department of Mechanical Engineering, University College London, London WC1E 7JE, United Kingdom

**Keywords:** Cardiovascular disease, Blood vessel remodelling, 3D Vascular models Biomaterials, Personalised disease modelling

## Abstract

Many cardiovascular diseases (CVD) are driven by pathological remodelling of blood vessels, which can lead to aneurysms, myocardial infarction, ischaemia and strokes. Aberrant remodelling is driven by changes in vascular cell behaviours combined with degradation, modification, or abnormal deposition of extracellular matrix (ECM) proteins. The underlying mechanisms that drive the pathological remodelling of blood vessels are multifaceted and disease specific; however, unravelling them may be key to developing therapies. Reductionist models of blood vessels created *in vitro* that combine cells with biomaterial scaffolds may serve as useful analogues to study vascular disease progression in a controlled environment. This review presents the main considerations for developing such *in vitro* models. We discuss how the design of blood vessel models impacts experimental readouts, with a particular focus on the maintenance of normal cellular phenotypes, strategies that mimic normal cell-ECM interactions, and approaches that foster intercellular communication between vascular cell types. We also highlight how choice of biomaterials, cellular arrangements and the inclusion of mechanical stimulation using fluidic devices together impact the ability of blood vessel models to mimic *in vivo* conditions. In the future, by combining advances in materials science, cell biology, fluidics and modelling, it may be possible to create blood vessel models that are patient-specific and can be used to develop and test therapies.

**Statement of significance**

Simplified models of blood vessels created *in vitro* are powerful tools for studying cardiovascular diseases and understanding the mechanisms driving their progression. Here, we highlight the key structural and cellular components of effective models and discuss how including mechanical stimuli allows researchers to mimic native vessel behaviour in health and disease. We discuss the primary methods used to form blood vessel models and their limitations and conclude with an outlook on how blood vessel models that incorporate patient-specific cells and flows can be used in the future for personalised disease modelling.

## 1 Introduction

Cardiovascular disease (CVD) is the leading cause of death worldwide [1]. One of the fundamental consequences of CVD is pathological remodelling of vascular tissues which can lead to complications such as aneurysms, myocardial infarction, ischaemia and stroke [2]. Studies using experimental animals and excised human tissues have proved invaluable in elucidating some of the mechanisms that drive pathological vascular remodelling. However, vascular dysregulation often involves multiple complex and dynamically interacting factors. As a result, confounding and/or redundant factors present in native tissues and whole experimental organisms can often hinder the discovery of the underlying mechanisms that govern these tissue-level changes, an understanding of which is likely essential in developing therapies.

Models of blood vessels built by combining cells with biomaterial scaffolds *in vitro* can serve as useful analogues to study some cell and tissue behaviours, in a reductionist manner [3]. By controlling experimental conditions, biological and mechanical factors can be introduced systematically, allowing study of how each impacts specific cell populations. Furthermore, such models can aid in reducing the use of animals in research [4]. *In vitro* models often rely on traditional 2D culture techniques; however, biomaterials, and in particular ECM-mimicking hydrogels may allow for more representative 3D cultures that are better able to recapitulate native cell behaviours in the 3D structure of the vessel wall [5–7]. Further more, advancements in the field of fluidics may also allow for the incorporation of fluid forces into models [8–11], which are vital in replicating the role of haemodynamics in driving vascular pathologies. In this review, we summarise some of the key considerations in developing *in vitro* models for vascular disease modelling by exploring the impact of biomaterials, culture methods and fluidic stimuli. We then explore how combining these factors may allow for the development of holistic models that can recapitulate the mechanisms of vascular disease progression. The purpose is to aid the reader in the design of 3D *in vitro* models tailored to study a range of vascular pathologies.

## 2 Designing engineered blood vessels

### 2.1 Pathological vascular remodelling

Mammalian vasculature is a highly dynamic tissue that constantly remodels throughout life. Balance is maintained through a tightly regulated network of complex inter- and intracellular behaviours regulated by biochemical and mechanical cues [2, 12, 13]. However, in disease, this intricate balance is often disrupted leading to vessel pathology. Causes of pathological remodelling can be classified into 3 main areas: changes in vascular cell phenotype, proliferation, or motility; degradation or modification of the extracellular matrix (ECM); and abnormal deposition of ECM [2]. These pathological changes can be driven by any combination of disrupted intercellular communication, changes in ECM composition, and/or abnormal haemodynamic stimulation of the vascular cells. Genetic factors potentially play a role in all of these mechanisms; however, discussion of this topic is omitted here.

**Fig. 1:**
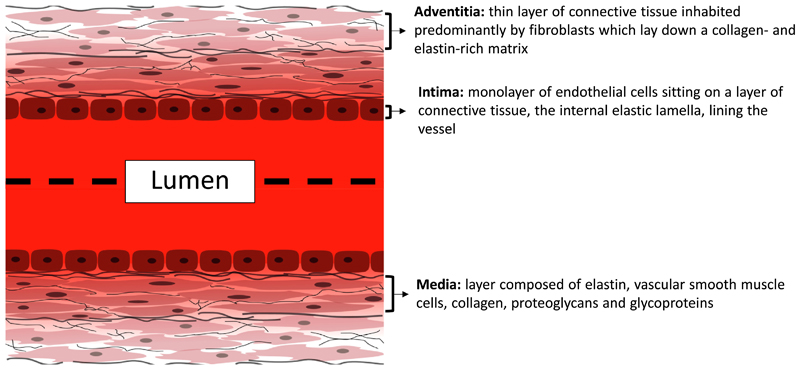
Simplified diagram of the arterial wall showing the 3 main tissue layers. Endothelial cells form a confluent layer called the intima, which is supported by structural extracellular matrix proteins. Directly underneath is the media, which contains vascular smooth muscle cells embedded within a matrix containing elastin and collagen fibres. The outermost layer of the vessel wall, the adventitia, contains fibroblasts in a collagen and elastin-rich matrix.

Arteries, veins and arterioles are composed of 3 main cell types, endothelial cells (ECs), vascular smooth muscle cells (VSMCs) and fibroblasts (FBs) arranged in 3 layers (Fig. 1). The outermost layer, the adventitia, is a thin layer of connective tissue inhabited predominantly by FBs which lay down a collagen- and elastin-rich matrix to provide vessel mechanical strength [14]. Next is the tunica media that gives the elastic properties of the wall and is composed of elastin, VSMCs, collagen, proteoglycans and glycoproteins [14]. Finally, the intima is made up of a monolayer of ECs sitting on a layer of connective tissue, the internal elastic lamella, lining the vessel lumen. By comparison, capillaries are made up of a single layer of ECs which line a basement membrane [15,16]. Broadly speaking, veins are thin-walled compared to arteries [16]. Veins tend to support lower flow pressures and velocities compared to arteries [16]; however the composition and thickness of each layer differs depending on location, even within the same vessel [14]. For the purposes of this review, we refer to large vessels as veins and arteries that contain 3 layers.

Pathological vascular remodelling includes inward remodelling, which can drive vessel occlusion, structural changes in which vascular diameter is not altered, and outward remodelling resulting in vessel wall weakening [2]. Inward remodelling of the vessel wall is predominantly associated with intimal thickening caused by an increase in cell number and ECM deposition towards the vessel lumen [17]. This occurs through a combination of migra-tion of cells from inside the vascular wall out into the lumen and recruitment of inflammatory cells. Such inward remodelling can occur in response to vessel injury and increased shearing forces due to the reduction of vessel calibre [18]. Outward remodelling is associated with loss of ECM proteins, cell death and ultimately weakening of the vessel wall. These changes in vessel morphology affect haemodynamics by disrupting the flow path. They also alter pressures, velocities, and flow profiles, both locally and systemically depending on the site. This aberrant vessel function has knock-on effects on the cardiovascular system as a whole causing flow insufficiency, regurgitations, increased frictional losses and pressure changes thereby increasing the overall cardiac burden, and in the worst cases, causing blockages or leaking of the system.

**Fig. 2:**
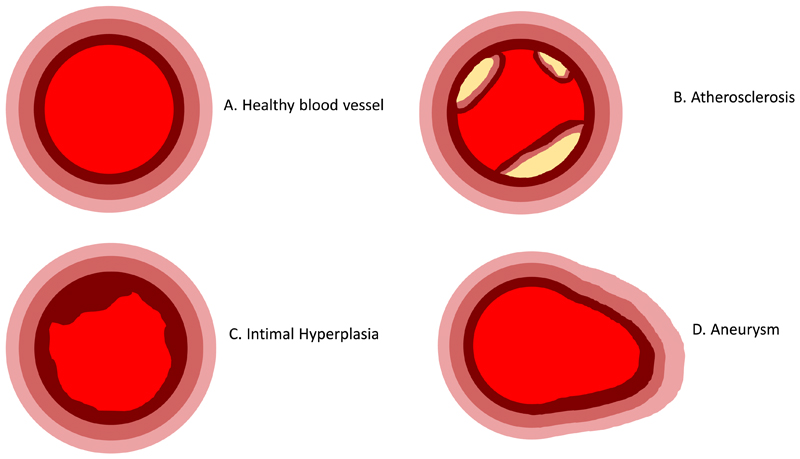
Schematic representation of common pathologies marked by abnormal remodelling of the blood vessel A) Healthy blood vessel in which 3 distinct tissue layers are observed. B) Atherosclerosis characterised by the accumulation of lipids and formation of plaques stabilised by fibrous caps. C) Intimal hyperplasia in which migration of vascular smooth muscle cells causes narrowing of the lumen. D) Aneurysm formation where the vessel diameter increases as the vessel wall becomes weakened and dilated.

One of the most common pathologies associated with aberrant vessel remodelling is atherosclerosis. Atherosclerotic plaques form through accumulation of lipids in the ECM, which prompts ECs to express adhesion molecules that can bind to and thus recruit leukocytes [19, 20]. This leads to a vicious cycle of lipid accumulation, inflammation and cell death. Plaque formation also drives the recruitment of VSMCs into the vessel lumen which form a fibrous cap that stabilises the plaque (Fig. 2). Intimal hyperplasia (IH) is another pathological condition that can lead to vessel narrowing. However, unlike atherosclerosis, IH is associated with migration of VSMCs from within the vessel wall out towards the lumen, where they form an occluding lesion [21]. Increases in vessel diameter, on the other hand, is a distinctive feature of aneurysms. Dilatation is caused by a mechanical failure of the vessel wall due to matrix dysregulation, and consequently the loss of structural proteins such as collagen and elastin [22, 23]. This is thought to be mediated by apoptosis of the VSMCs, which are responsible for depositing ECM proteins, and over-production of matrix degrading matrix metalloproteinases (MMPs), predominantly by VSMCs and FBs, which destroys ECM components that are vital for vessel tone and cell attachment [24–27].

### 2.2 Biomaterial scaffolds in in vitro models of the vasculature

In engineered models of blood vessels, a key component is choice of scaffold material. The scaffold should support physiologically relevant cellular arrangements and be able to mimic tissue behaviours. Amongst other requirements, scaffolds should support long-term cell culture, allow the diffusion of nutrients and solutes involved in intercellular communications, and permit ECM remodelling. The intrinsic properties of the scaffold, such as its stiffness and the presentation of adhesion sites, should also allow for the maintenance of physiologically relevant cell phenotypes. Furthermore, as the material structure impacts interactions between embedded cells and applied mechanical forces, the biomaterial should both be able to withstand applied strain and stresses, but also impart mechanical cues to cells in a physiologically realistic and characterisable way.

Given the requirement of the matrix to deliver complex cues to embedded cells in a controllable manner, identifying an appropriate 3D material is not trivial. However, advances in materials science have produced platforms that can support cell survival, adhesion, and importantly, interaction with their environment, allowing cells to actively remodel and ultimately replace the matrix surrounding them [5, 6, 28]. Such materials are not only important in supporting long-term culture, but also in establishing disease models driven by matrix dysregulation, as they enable the study of how cells interact with their surrounding ECM.

Hydrogels are suitable for 3D cell encapsulation due to their two-phase composition with a solid polymer phase mimicking the ECM protein scaffold and a surrounding liquid phase available for transport of nutrients [7]. The first choice is between naturally derived or synthetic gels. Naturally derived gels offer an advantage as they contain natively occurring ECM proteins, and thus can recapitulate native tissue cues. Collagen gels are one of the most commonly used naturally derived hydrogels due to their high degree of biocompatibility, ability to support cell adhesion, availability, ease of use and ability to produce stable cross-linked networks [29]. Collagen is also the most abundant ECM protein in the media and adventitia [30], and so collagen gels, particularly formed from collagen type I, have been extensively employed in vascular studies [31,33–35]. Indeed, the ease with which cells can interact with naturally derived proteins has allowed important insights into cell matrix interactions. For example, rat aortic SMCs, adventitial FBs and myofibroblasts encapsulated in rat tail tendon-derived collagen type I migrate through their surrounding matrix by upregulating MMP-1 production [33].

However, cells’ interactions with native matrices have also been shown to modulate phenotype. Indeed, whilst collagen type I is abundant in vascular tissues, alone it may not support correct VSMC phenotypes whose regulation is mediated through complex interactions with many different proteins [28]. On the other hand, Matrigel, which is derived from a murine sarcoma and is abundant in basement membrane proteins, provides cells with a multiprotein environment and is also often used for 3D cell culture. However, a comparison between VSMC behaviour in 3D culture using collagen type I and Matrigel revealed that, whilst proliferation of cells in Matrigel was slower, expression of typical phenotypic markers was progressively lost over time in both matrices [36]. This may be because batch-to-batch variability in Matrigel [37], and the presence of myriad undefined soluble factors and proteins may impact cell phenotype. For example, collagen type IV isolated from Matrigel can also present encapsulated cells with bound TGF*β*, which can regulate cellular responses [38]. Furthermore, these naturally derived hydrogels often lack the mechanical strength to withstand blood vessel pressures, and thus may not be suitable to deliver physiological mechanical cues to cells [35, 39]. In short, biologically derived matrices offer advantages in terms of delivering biocompatibility and bioactivity with relative ease. However, these often lack mechanical strength, and it can be difficult to control how and which specific biomolecules are presented to encapsulated cells.

Synthetic gels are an alternative which may provide tighter control over matrix properties than biologically derived gels, with clearly defined properties and hence more inter-sample consistency. Synthetic hydrogels are often formed by cross-linking polymer macromers either via photo-polymerisation or through chemical or physical cross-linking, creating a lattice-like structure [40]. The dimensions of the lattice can be modulated by altering the polymer chain length and thus the mesh network size. This allows control over the diffusion of solutes through the gel. Moreover, changes in hydrogel structure alter flow passing through the gel, and by extension the shear it imparts on encapsulated cells. Control over these features is an advantage for recapitulating 3D mechanical and biochemical cues in a reproducible manner.

A major drawback of synthetic gels is their innate bio-inertness, and therefore sites for cellular interactions must be incorporated to make them hospitable for cells. This can be facilitated in synthetic gels by inclusion of bioactive peptides [40, 41], which allow for control over the density and type of biological cues. Polymers can be modified to include peptides that contain the binding sites of native matrix proteins such as RGD, which mimics the fibronectin binding domain, as well as MMP-cleavable sequences, which allow cells to remodel and migrate through the 3D matrix. Inclusion of adhesive and degradable peptides can be tuned independently from matrix concentration, and by extension, stiffness, allowing orthogonal control of mechano-regulatory and biological cues [5].

Although not suitable for cell encapsulation, lactide and glycolide polymers have been proposed for blood vessel applications as they degrade by hydrolysis, and can be manufactured with high porosities, allowing for nutrient exchange [42]. Indeed, Polyglycolic acid (PGA) scaffolds have been shown to be replaced by SMC-derived ECM proteins, rendering the graft able to withstand pressures above 100-200 mmHg [43]. However, whilst PGA may improve upon the mechanical properties of collagen, studies have shown that PGA alone does not provide sufficient long-term mechanical strength unless progressively reinforced by native matrix synthesis [42]. Addition of other polymers to PGA such as poly-4-hydroxybutyrate (P4HB), poly-(lactic-co-glycolic acid) (PLGA), poly(L-lactic acid)(PLLA), poly(caprolactone) and polyethylene glycol (PEG) have been shown to improve their mechanical strength [42, 44–47].

**Fig. 3:**
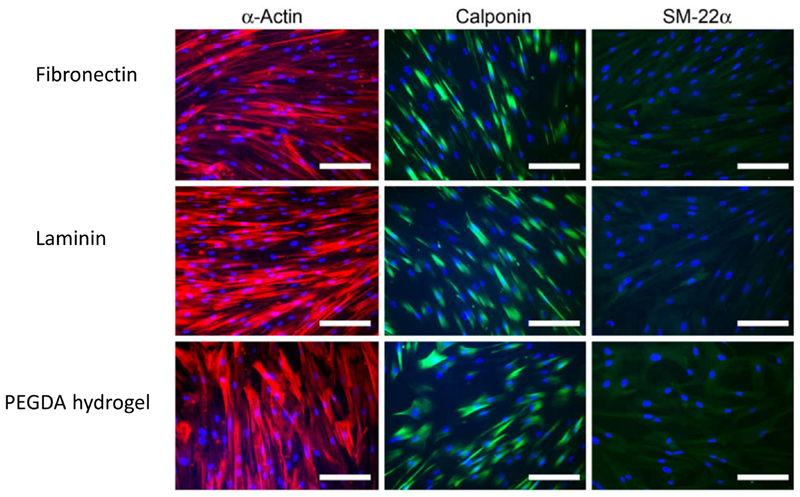
Fluorescence staining of human coronary artery smooth muscle cells for alpha-actin, calponin and SM-22a protein after 6 days of culture on fibronectin, laminin and a RGD-bearing poly(ethylene glycol) diacrylate (PEGDA) hydrogel, cultured in low serum (2% FBS) and containing heparin (400 mg/ml) to induce differentiation. Scale bar=200 μm. RGD peptide-containing gels support equivalent levels of expression of the contractile markers compared to culture on fibronectin or laminin, indicating that RGD-modified PEGDA gels can support differentiation of SMCs toward a contractile phenotype. Figure adapted from [28].

PEG has been identified as a suitable stand-alone material for vascular cell scaffolds due to its biocompatibility, hydrophilicity, resistance to protein adsorption, non-thrombogenic properties, high elasticity, and overall highly tunable properties [28, 48–53]. PEG gels allow efficient and controllable diffusion of biomolecules. The diffusivity of species can be controlled by altering the length of the polymer chains in the PEG macromer, and by extension the mesh network size [53]. PEG gels are also amenable to incorporation of bioactive peptides through, for example, Michael-type addition reactions in which peptides are presented pendantly or used as cross-linkers between the PEG macromers [5, 54, 55]. Indeed, peptide-modified PEGDA hydrogels containing the RGD sequence are able to support the differentiation of human coronary artery SMCs toward a contractile phenotype equally well as when they are cultured on fibronectin or laminin [28] (Fig. 3).The high characterisability of PEG gels and thus control over important features, including diffusion of soluble factors and cell-matrix interaction, makes PEG-based gels especially suited to disease models designed to answer mechanistic questions. Furthermore, tight control over the matrix properties can also be crucial when incorporating other stimuli such as flow, enabling reliable prediction of the response of the matrix to mechanical force.

As both naturally derived and synthetic gels have drawbacks, it is also possible to create hybrid materials which combine the advantages of each [56–59]. For example, gelatin, collagen and elastin have been combined with synthetic poly(ε-caprolactone) to increase scaffold tensile strength compared to gelatin or collagen and elastin alone [57]. Furthermore, photocrosslinked gelatin methacryloyl (GelMA) in which naturally derived gelatin is modified with methacrylic anhydride has also been used to create vessel-like structures with separate layers encapsulating rat aortic SMCs and 3T3 fibroblasts and lined with a human umbilical vein endothelial cell layer [60].

### 2.3 Incorporating vascular cells: cellular arrangements and interactions

Blood vessels must withstand high pressures from fast blood flows whilst remaining compliant and adaptable to changing supply needs [61–63]. Regulation of vessel function is mediated through the contraction of the VSMCs, as well as composition of the ECM in each layer, and therefore is a combination of the passive structural and active cellular behaviours of the vessel. In disease and during vessel remodelling, VSMCs can change phenotype from that of a contractile cell to a “synthetic cell”. These cells lose their differentiated contractile properties and can migrate, proliferate and modify the ECM. For example, in aneurysms, loss of structural proteins in the medial ECM renders the vessel weak, making it prone to ballooning and rupture [64]. Conversely, in atherosclerosis, over-production of collagens and proteoglycans by VSMCs lead to areas of lipid accumulation, driving inflammation, necrosis and the formation of plaques, which encroach on the lumen, causing vessel stenosis [65].

*In vitro* setups for disease modelling should ideally recapitulate conditions that foster correct ECM maintenance to study the potential role of their disruption in disease progression. Indeed, at baseline, models should promote healthy cell function with the ability to monitor biomarkers which signal pathological behaviours. The geometry of the vessel model and choices in seeding methods determine the scope for physiological cell phenotypes, morphologies, and intercellular interactions. Next, we present a review of different *in vitro* setups, and a discussion of the key design choices impacting the fidelity of *in vitro* culture systems. In particular, we explore the impact of cell-ECM interactions and intercellular communication on maintaining correct cell phenotypes.

### 2.4 2D versus 3D culture

Culturing cells on tissue culture plastic reduces complex 3D tissue structures into simplified cell monolayers. Whilst this has allowed useful observations, cell behaviour in 2D is often markedly different from that *in vivo* [66, 67]. Discrepancies between cell behaviour in 2D and 3D are not simply due to a change in dimensionality, but are rather also attributable to downstream impacts [68]. For example, in a 3D environment, cells are presented with adhesion sites in all directions, which alters their morphology [66, 68]. Cells sense their environment through adhesions with their ECM. Thus, stiffness and mechanical stress are felt differently by cells in 3D than in 2D. The makeup of the ECM in which cells are surrounded is also a governing factor in directing cell phenotype. Indeed, cells respond differently to different ECM proteins in 2D compared to 3D [31]. Furthermore, transport of nutrients and soluble biomolecules is also altered in 3D, and therefore, studying the effect of disequilibrium of any of these factors is likely to be more faithful to that *in vivo* if studied in 3D. Importantly, whilst 2D endothelial cell culture may be appropriate due to their native monolayer presentation, VSMCs and FBs, which normally reside in an ECM-rich 3D structure, are known to be regulated by interactions with their 3D matrix [31,44, 69–72].

VSMC-ECM interactions are an important regulator of vascular cell adhesion, migration, proliferation and phenotype [73]. Indeed, VSMCs lie on a phenotypic spectrum from synthetic to contractile and express different markers accordingly. Whilst distinction between contractile and synthetic cells is not always clear, in a healthy mature vessel the majority of cells are contractile. In a contractile state, VSMCs reinforce the vessel wall, enabling it to withstand fluid pressures. This is achieved through expression of dystrophin-glycoprotein complexes, which link actin filaments within the cell’s cytoskeleton to the surrounding ECM [74]. Conversely, VSMC transdifferentiation or reversion to a synthetic state has been implicated in ECM dysregulation and disease progression [75]. Indeed, Chen *et al.* have shown that a subpopulation of VSMCs may themselves contribute to aneurysm formation by transdifferentiating into mesenchymal cells in response to loss of TGF*β* signalling. Accumulation of these mesenchymal cells in the aortic wall of Apoe^-/-^ mice on a hypercholesterolemic diet results in loss of the contractile VSMC and accumulation of lipid deposits and ossifications, which together contribute to aneurysm formation [76].

The presence of specific binding motifs and cellular engagement and disengagement with adhesion sites have been shown to govern VSMC phenotype [31, 44, 69–72]. Fibronectin and collagen type I have been implicated in driving the synthetic phenotype, whilst laminin and collagen type IV, promote the contractile. These relationships have been confirmed in disease models, such as atherosclerosis, where VSMC phenotypic changes are known to be mediated through interactions with fibronectin [77, 78]. Given that the VSMC synthetic phenotype and ECM makeup are both markers of pathology, cell-ECM interactions and matrix composition should therefore be considerations in designing *in vitro* models. Furthermore, VSMC’s response to ECM proteins differs in 2D and 3D setups. In a study using rat aortic SMCs, cells were cultured on a 2D collagen type I surface or embedded within collagen type I gels at the same polymer concentration and compared to cells cultured on tissue culture plastic [31]. Embedding the cells led to a downregulation of smooth muscle actin (SMA), a marker of VSMC contractility, as well as to a decrease in cell proliferation compared to the 2D collagen condition and tissue culture plastic controls. Levels of SMA and proliferation were no different in 2D collagen cultures compared to tissue culture plastic controls, leading the authors to conclude that the changes in cell phenotype were independent of matrix composition and could be attributed to cell response to 3D culture.

When VSMCs are isolated from tissue samples, they often lose their phenotype, with expression of relevant markers dropping with successive passages [31]. These studies highlight that both protein presentation and dimensionality impact VSMC’s ability to retain contractile phenotypes. This highlights the importance of studying VSMCs in 3D environments to recapitulate *in vivo*-like responses.

### 2.5 VSMC-EC interactions

Despite its important role in regulating phenotype, VSMC interactions with ECM proteins alone may not be sufficient to retain VSMC contractility. Indeed, intercellular communication between VSMCs and ECs also regulates VSMC behaviours, including proliferation, quiescence, morphology, and by extension vasodilation and healthy vessel maintenance [67, 79–88]. Co-culture of VSMCs with ECs has been shown to regulate SMC phenotype [87–89] and to prompt VSMCs to adopt a more contractile-like state, which makes including co-cultures in blood vessel models an important consideration.

The endothelial layer creates a selectively permeable barrier to the vascular wall by controlling transport of molecules and immune cells as well as by facilitating cell-cell communications through adherent junctions, tight junctions and gap junctions [90, 91]. VSMC and EC communicate with one another both through direct cell-to-cell contact and through diffusion of secreted molecules [92–94]. Disruption of the endothelial barrier is thought to be the first step in the formation of atherosclerotic plaque [95]. For example, SMC proliferation in rat carotid arteries was studied by denuding the endothelium, both with and without causing damage to the media [96]. Here, denudation alone was sufficient to drive lesion formation, highlighting the role of the endothelial layer in vascular pathology. Such findings have been confirmed in *in vitro* injury models demonstrating how disruption of the EC layer can stimulate proliferation of the underlying VSMCs [97].

**Fig. 4:**
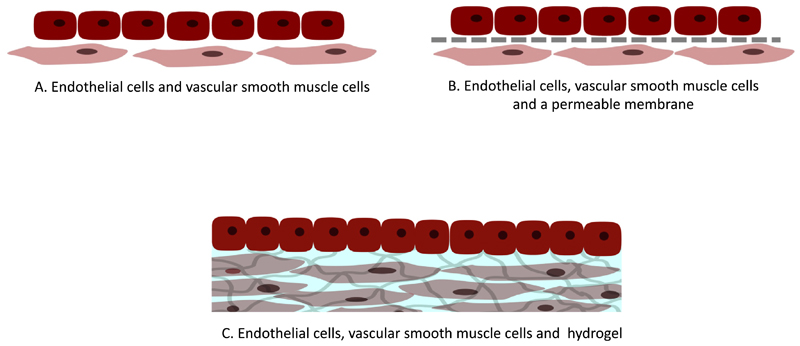
Schematic representation outlining experimental setups for co-culturing endothelial cells with vascular smooth muscle cells. A) Culture of endothelial cells directly on top of vascular smooth muscle cells. B) Separation of both cell types by means of a permeable membrane. C) Culture of endothelial cells on 3D gels containing vascular smooth muscle cells.

Maintenance of a healthy endothelium is therefore vital in a healthy vessel, and one key factor is facilitating correct EC regulation of VSMCs in a feedback loop. These cells communicate via gap junctions which require cell-cell contracts, and therefore culture systems which allow proximity may have advantages in retaining correct VSMC phenotype. There are 3 main ways in which co-cultures have been reported: culture of ECs directly on top of VSMCs [98, 99], separation of both cell types with a permeable membrane [1, 79, 82, 86, 97], and culture of ECs on 3D gels containing encapsulated VSMCs [85,99] (Fig. 4). The main difference in each of these methods is the proximity that the different cell types have to each other, and the inclusion (or not) of a 3D cell niche for the VSMCs. The distance between cells impacts their ability to create gap junctions and hence the way in which they can interact. Furthermore, given that communication is also dependent on diffusion of soluble factors, the distance between the cells and the diffusivity through the layers may be key [100,101].

Taken together, these observations suggest that disease modelling should optimally start with a 3D culture with multiple cell types that allow for intercellular communication. In tissue engineered grafts that aim to replace whole sections of pathological tissue, these factors are often taken into account as the grafts are required to adopt the full function of vessels once implanted. Therefore, advances in tissue engineering have also provided important insights into the development of artificial layered constructs, which could be useful for disease modelling. The first engineered 3D vessel was created by Weinberg and Bell in 1986. In their model, a 3-layer construct composed of collagen gels laden with FBs and bovine VSMCs was created with a confluent monolayer of ECs lining the structure [35]. Whilst many physiological aspects of the native vessel were recapitulated with this model, it suffered from limitations in terms of vessel resistance to pressure as well as in the composition and morphology of the layers, as it lacked the elastin natively found in the intima.

Subsequent studies have improved on this original work by using materials with greater control over matrix properties, by improving manufacturing techniques, by including mechanical loading to simulate physiological strain, and importantly, as later discussed, by incorporating fluid force stimulation [43,45,48,102–106].For example, Niklason *et al*., created an artificial vessel by seeding bovine VSMCs on tubular biodegradable PGA scaffolds, which they cultured under pulsatile flow and in the presence of factors to support collagen synthesis and ECM protein cross-linking [43]. This created a graft with a smooth surface onto which EC could adhere, with the final structure able to withstand physiological pressures. Indeed, when implanted into a miniature swine model, the construct developed a highly organised structure with minimal signs of inflammation compared to a xenograft control.

In summary, the structure and composition of 3D vascular models will influence interactions between vascular cell types and with their ECM. Therefore, how these components are assembled in an *in vitro* model will impact its ability to recapitulate the native tissue’s morphology, and its efficacy in mimicking native-like cell migration, proliferation, and ECM secretion/degradation, and by extension, vascular pathologies. Therefore, careful consideration is warranted in balancing simplicity against workability in the model to effectively capture particular aspects of disease or disease pathogenesis.

### 2.6 Incorporating mechano-stimulation: stress and strain

**Fig. 5:**
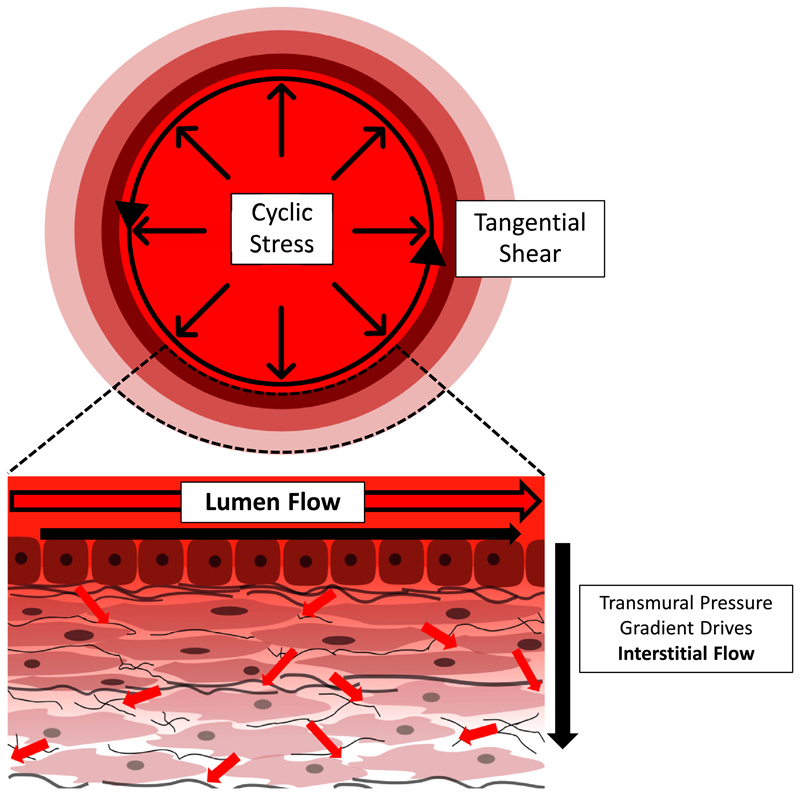
Schematic of the 3 main axes of mechanical stimulation by flow in large vessels. Cyclic strain is developed in the wall as pulsatile flow develops normally, applying pressure forces that compress the vessel wall. Luminal shearing of endothelial cells is caused when blood flow passes over the cells. Finally, transmural pressure gradients drive interstitial flow through the vessel wall, which applies shear forces on embedded cells. Red arrows depict movement of flow through the lumen and interstitially in the vessel wall.

Blood flow is pulsatile in nature, as pressure and velocity vary as the heart ejects blood and refills [107]. Haemodynamics within the system are determined by a combination of vessel geometry, driving pressures that force blood round the system, and the material properties of both vessels and blood itself. Therefore, blood flow profiles vary temporally and spatially along the circulatory system. Arteries and veins are exposed to continuous mechanical flow-mediated stimulation through two main mechanisms: deformation of the vessel wall due to blood pressure, and fluid shearing forces [62, 108]. Pressure from the pulsatile flow acts normally to the vessel wall, delivering cyclic strains through circumferential stretching of the tissue [108] (Fig. 5), which the vessel wall resists by developing stresses [107]. Direct frictional shearing forces, on the other hand, act tangentially on the cells as a result of viscous flow passing over and around them [108,109] (Fig. 5).

The majority of blood flow passes through the lumen and delivers a shearing force to the ECs lining the vessel. ECs are highly mechanosensitive to blood flow cues, which they sense through their cytoskeleton as the cell deforms in response to fluid forces [110]. Indeed, luminal shear is a key regulator of vessel tone, prompting adaptive dilatation of the vessel wall [111–114]. It also regulates cell morphology, proliferation, protein expression and importantly for atherosclerosis research, the ability of ECs to attract monocytes [114–116]. ECs achieve regulation of the medial wall by producing vasoconstrictors and dilators, and in healthy vessels, maintain a non-adhesive lumen that has antithrombotic and anticoagulant properties [117]. They also communicate to the matrix synthesising cells embedded in the wall below [93, 118–120].

In pathologies in which the vessel diameter is changed, healthy blood flow patterns are altered, and by extension, so are the mechanical forces imparted on the constituent cells. Given the crucial regulatory role of blood flow, it is therefore not surprising that pathological endothelial function precipitated by abnormal haemodynamics has been highlighted in many vascular pathologies [63,121,122]. Much effort has therefore focussed on understanding the mechanisms by which lumen flow disrupts endothelial function and its implications for disease progression. These include, but are not limited to, shear stress-driven development of atherosclerosis [63, 123] and IH [124]. Moreover, flow eccentricity and high wall shear stress have been linked to dilatation in bicuspid aortic valve disease [125–128].

In addition to the luminal flow, transmural pressure gradients between the lumen and the outermost layer of the aortic wall drive a small amount of flow interstitially around the VSMCs and FBs (Fig. 5). The effect of this interstitial flow is often neglected due to slow flow velocities in this region (~10^−6^ cm/s) [129]. However, due to the small interstitial spaces in the media wall, although flow is slow, the resultant forces have been shown to be non-negligible [129]. Using an analytical model, these velocities have been estimated to result in a shear stress of ~1 dyn/cm^2^ [129]. Furthermore, in the case of injury or disease in which the luminal layer becomes disrupted or the ECM composition changes, the permeability of the EC layer may increase, exacerbating this effect [118,130,131]. *In vitro* studies have revealed that interstitial flow is able to impact SMC and FB migration, proliferation, and survival; as well as SMC alignment, contraction, phenotype and signalling [118]. It may therefore be important to include flow effects both luminally and interstitially in vascular models. Recapitulating *in vivo* interstitial stresses on VSMCs and FBs will likely require the use of 3D constructs. The structure of the interstitial tissue, driving pressure gradient, and the cellular arrangement inside the wall determine the nature of the flow, and therefore, 2D setups are unlikely to account for these effects. Indeed, with VSMC and FB dys-regulation heavily implicated in vascular pathology, inclusion of 3D flow in disease modelling may be essential. Therefore, we next discuss models to recapitulate the 3 main axes of stimulation: cyclic stress, lumen stresses and interstitial stresses.

### 2.7 Providing mechanostimulation in in vitro models

**Fig. 6:**
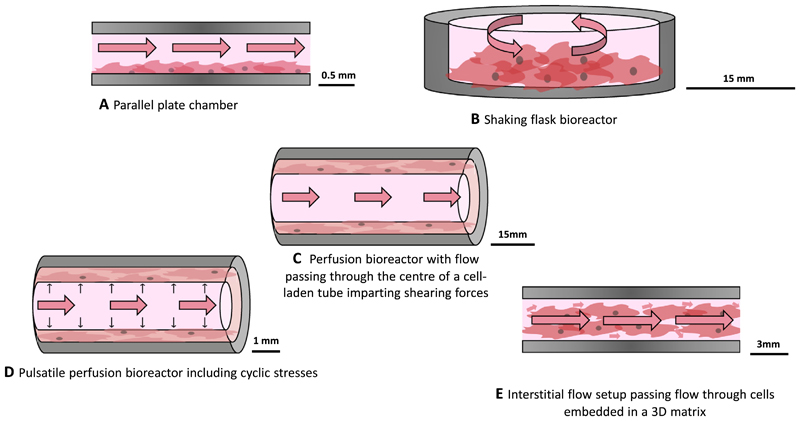
Schematic of different bioreactor setups showing the movement of flow in each (pink arrows) A) A flat parallel plate chamber in which flow is passed over monolayers of cells delivers shear stresses to cells cultured along the wall. B) A shaking flask bioreactor in which the cell culture is rotated to stimulate motion of the cell culture media above imparts shear stresses on the cells. C) A perfusion bioreactor with flow passing through a central tubular channel surrounded by cells cultured in 2D or 3D applies shear to the cells in contact with the moving media. D) By incorporating pulsatile flow into a perfusion bioreactor, cells experience strain both from normally acting pressure forces as well as tangential shear. E) A parallel plate bioreactor can also deliver flow directly through a 3D matrix containing embedded cells, stimulating them with interstitial shear.

Flow stimulation is imparted on cell cultures *in vitro* using fluidic devices known as bioreactors or flow chips, in which cells are placed in the path of flow (Fig. 6). Design criteria of these constructs are determined by considering the physiologically relevant forces to impart, and by extension the properties of *in vivo* flow to be recapitulated, as well as practical laboratory constraints.

The vascular system contains flows of different scales and regimes dependent on the size, geometry and location of a vessel. The distinction is made between macro and micro-scale flows and the relative contributions of inertial, viscous and pulsatile forces. These factors can be quantified numerically using the Reynolds and Wormsley numbers, dimensionless parameters calculated using flow velocities and vessel dimensions which provide the ratio of inertial to viscous forces, and pulsatile to viscous forces, respectively [15, 107]. The Reynolds number also evaluates whether the flow is laminar or turbulent. In microvessels, viscous forces dominate, and the pulsatile nature of aortic flow is dampened, whereas in the macro circulation inertial terms become dominant with Reynolds number ranging from 1 in small arterioles to approximately 4000 in the aorta [107]. Pulsatile flow is highly relevant for the aorta and arterial components of the macro circulation due to the cyclical beating of the heart [15,107]. Pressure variation along the vessels and throughout the cardiac cycle also varies the circumferential strain as the vessel stretches in response.

Inevitably, the full complexity of *in vivo* flow is exceptionally challenging to capture *in vitro*, so careful consideration must be made as to the balance of building a system which is limited by over-complexity, versus careful tuning of specific flow parameters. Mechanical shear and strains are dependent on many different parameters and so there is an opportunity to control design criteria to provide controlled flow characteristics. The properties of the flowing medium as well as the geometry and substrate over or through which the fluid is moving influence the mechanical shear as flow passes over ECs and around embedded VSMCs. Pressures resulting from high flow velocities compact the tissue circumferentially, with the resistance of the matrix determining the degree of deformation. In the case of shearing forces, the magnitude of the mechanical force is dependent on the flow profile as well as the viscosity of fluid determining the friction between the flow and the vessel wall or interstitial matrix.

Determining exact values for these forces *in vivo* is challenging. Shear and strain cannot be directly measured *in vivo*. Instead, estimations are made using imaging techniques or patient catheterisation, often extrapolated or interpreted using mathematical models. For example, estimates of local velocity gradients are obtained using imaging techniques such as Doppler echocardiography or MRI, and these are then used to infer values of shearing force [132– 134]. However, capturing all the intricacies of the full flow profile is challenging due to resolution limitations, and in particular estimates near the wall are hindered by difficulties in accurately tracking the moving vessel boundaries [132]. Furthermore, the assumptions required to apply mathematical models to these data can often simplify the complex *in vivo* environment. Nevertheless, advances continue to improve the robustness and accuracy of these estimates by improving the detail with which velocities are captured and the complexity of the models used [133, 135].

When translating *in vivo* mechanical stimuli into *in vitro* systems, concessions in terms of operating medium, scale and geometry must be made. A major difference in *in vitro* models versus *in vivo* is the use of cell culture medium in place of blood. Blood is highly viscous, with a complex rheology due to suspended particulates, and so even in larger vessels, blood flows impart high shear stresses. To recapitulate this *in vitro* with culture medium, faster flows or increased spatial velocity variability is needed to maintain the same flow resistance, and there is a compromise to be struck balancing viscosity, flow velocity and geometry to reach physiological levels of shear. Importantly, as balancing these factors determines the regime of the flow, careful consideration must be made when changing flow velocities and chamber dimensions to ensure flow is representative of the pathology to be studied. By passing flow through smaller channels, the spatial variation in velocity is increased, and therefore with slower or less viscous forces, high levels of shear can still be achieved.

Furthermore, practical considerations in terms of scale may have to be made with most laboratory setups by sizing down. Microfluidics (devices with largest dimension of order 1mm) offers many advantages, including reducing the number of cells, amount of reagents needed and enabling high throughput [8–11, 136–138]. Furthermore, with increasing ease of small-scale manufacture, microfluidic devices are able to impart specific mechanical cues with high tolerance [137]. Importantly, the smaller dimensions of microfluidic devices ensure flows remain laminar [10], which may be advantageous as fluid behaviour is more easily characterised and predicted in laminar regimes [10]. However, this could be a drawback for recapitulating pathological flow at higher Reynolds numbers.

Though predominantly used for the study of microvasculatures, microfluidics illustrate that spatial complexity can be introduced when using small scale flow chambers. Indeed, as physiological flow is highly heterogeneous, microfluidic systems lend themselves to precise patterning of fluid pathways [10]. Development of micro-physiological models to study whole organ pathology are often used to create “organ-on-a-chip” models [139–142]. To this end, microfluidic chambers have been designed not only to impart fluid forces to cells, but also to create microvascular networks for disease modelling and drug-screening assays [143–145]. For example, PDMS chambers combining cancer cells and ECs embedded in matrix surrounded by microfluidic channels have been designed to deliver arteriole and venule flow regimes [144]. Here, ECs migrate out to the fluid channels, eventually lining and sealing them to create a lumen through which flow passes. Then, by incorporating different cancer cells, a vascularised microtumour model can be developed. Studying tumours in the presence of vasculature can not only be used to model tumour development and response to drugs, but also cancer metastasis, through a process called extravasation by which cancer cells move through the endothelium and into the bloodstream [146, 147].

Using cancer cell extravasation models, human mammary adenocarcinoma cells movement has been shown to be impacted by the surrounding matrix. Indeed, osteogenic-cell conditioned gels promote more movement than un-treated collagen type I gels, thus highlighting the impact of the tissue environment in cancer progression [148]. However, such devices model the blood vessel wall as a monolayer of ECs, and therefore can fall short in representing the complex vascular networks surrounding tumours *in vivo*. To improve on these studies, microvasculature models developed for organ-on-a-chip tumours can be incorporated into extravasation studies [149]. For example, micro-vasculatures have been formed by co-culturing primary human bone marrow-derived stromal cells differentiated towards the smooth muscle cell lineage with ECs. This resulted in more physiologically relevant blood vessel structures compared to those formed with ECs alone [150]. Moreover, using this model, the authors were further able to explore the impact of shear force on extravasation by passing flow through the vascular network, resulting in wall shear stresses of 0.25 dyn/cm^2^ on ECs. The presence of flow decreased the permeability of the vasculature by tightening cell-cell junctions between the ECs. This in turn impacted extravasation rates, which were lower in flow conditions.

However, despite many advantages of using small scale devices to apply shear to cells, mircofluidics are not necessarily more physiologically representative [10]. Use of macro scale bioreactors should thus not be disregarded, particularly when studying vessel pathologies in larger vessels. Furthermore, when recapitulating large vessel events in which the Reynolds number is typically higher than for flows in microfluidic devices, fidelity to the flow regime may be important depending on the mechanism driving the shearing forces.

### 2.8 Towards the complete recapitulation of all factors

Thus far, we have highlighted the importance of co-culture systems within 3D materials, and fluid flow that captures all 3 axes of stimulation to recapitulate important aspects of blood vessel structure and the stimuli that act on it *in vivo*. Whilst models incorporating all these aspects combined do not yet exist, there are examples in which these factors have been incorporated individually using step-by-step approaches (Table 1).

A number of methods have been described to pass flow over monolayers of cells, including passing flow in a channel above the cells or shaking culture flasks [151–156]. Using such setups, 2D cultures of VSMC exposed to shear have been shown to upregulate expression of TGF*β* [156], and downregulate MMP-2 expression, which inhibited cell migration [157]. Similarly, 3-25 dyn/cm^2^ of shear stress have been shown to prompt increased VSMC secretion of FGF-2 [155]. Some studies have also combined shear stress with pressure and stretch by seeding EC on a thin concave polymer film which was stretched to mimic pulsatile flow [158]. The authors were able to show that their device was able to deliver both physiological and pathological pressure, flow, strain, and shear stress whilst allowing cells to align correctly and maintain barrier in-tegrity compared to static cultures. Nevertheless, the shear stress levels on VSMCs in many studies are often higher than normal physiological levels and can be more than what cells are exposed to, even in the case of extreme pathologies [159].

There have also been efforts to incorporate cell-cell interactions into blood vessel models stimulated with flow. For example, in [160] the authors separated co-cultures of ECs and VSMCs using a medial layer containing a variety of adhesion proteins that were exposed to flow using a parallel plate flow chamber. Similarly, Van Engeland *et al*. combined ECs with embedded VSMCs to create an “artery-on-a-chip” device [1]. In this setup, ECs and VSMCs were seeded in a culture channel separated by a membrane intended to simulate the porous internal elastic lamina found between these cell layers *in vivo*. This model allows for cell-cell contact, whilst simultaneously exposing them to physiological luminal shear stresses, and was able to support ECs and VSMCs to maintain expression of phenotypic markers and normal cell morphologies. In particular, ECs expressed higher levels of von Willebrand factor, and VSMCs expressed more SMA when cultured in the device compared to in 2D. However, in this model, VSMCs were cultured in 2D and interstitial fluid flowing through the 3D matrix was not taken into account.

Simulation of fluid stresses can also be achieved using perfusion bioreactors. Here, 2D or 3D cell cultures are seeded in annular structures and flow is passed through a central lumen [4, 43, 161– 165]. Such setups typically focus solely on shear; however, they have also been modified to pass flow through a 3D VSMC-seeded tubular structure with a surrounding channel, allowing the setup to then be pressurised to deform and thus strain the culture [165]. This allows independent control of circumferential stress and shear stress. Whilst this type of setup allows for study of fluid stresses and strains on 3D cocultures, transmural flow was not taken into account. Indeed, this component is often overlooked due to underestimation of the resultant mechanical forces on the inter-mural cells.

Although limited, there are a few studies that have incorporated interstitial flows into blood vessel models. Strategies to achieve this generally involve the culture of VSMCs and/or FBs in a 3D matrix across which a pressure gradient is applied to drive flow through the gel [32,33, 152]. For example, Wang *et al*. perfused a SMC-laden collagen gel by applying hydrostatic pressure and found that encapsulated SMCs upregulated production of prostaglandins compared to 2D controls under the same level of shear [32]. Using a similar approach, Shi *et al*. studied cell migration within 3D cultures to model neointima formation. They combined vascular FBs, myofibroblasts, and SMCs and found that motility was driven by upregulation of MMP-1 in interstitial flow compared to no-flow conditions [33].

In studies that apply interstitial flows, the forces imparted on the cells are often estimated using laws that govern flow through porous media by considering pressures applied across cultures and the average flow velocities through the matrix. As the relationship between velocity and stress is more complex for flow through a matrix compared to tangential shear, approximations are often made assuming average velocities and homogenous matrix properties throughout the 3D structure. However, whilst these approximations have served to good use, their underlying assumptions (which often treat the matrix as a bulk material) may oversimplify the spatial variation in shear acting on embedded cells. Estimates which consider the finer structural heterogeneity may give more realistic predictions of the forces experienced by the embedded cells. Synthetic materials in which matrix properties can be more robustly predicted may lend themselves more easily to these quantifications. We therefore suggest that advances in biomaterials may allow for more accurate predictions of 3D flows within the structure.

Furthermore, pathological flow has been implicated in tissue dysregulation, prompting cells to remodel their surrounding ECM. This includes excessive destruction of the tissue in aneurysm and abnormal luminal deposition in atherosclerosis. The composition of the ECM in turn alters the interstitial fluid mechanics, affecting the transport of solutes to embedded cells. Systems in which this interplay between matrix changes in response to mechanostimulation and the subsequent altering of these mechanical cues may be important to capture these bi-directional effects [166, 167]. Therefore, advances in biomaterials, which allow for representative cellmatrix interaction in the presence of flow is vital, once again highlighting the role of material properties for both meaningful static and dynamic disease modelling.

**Fig. 7:**
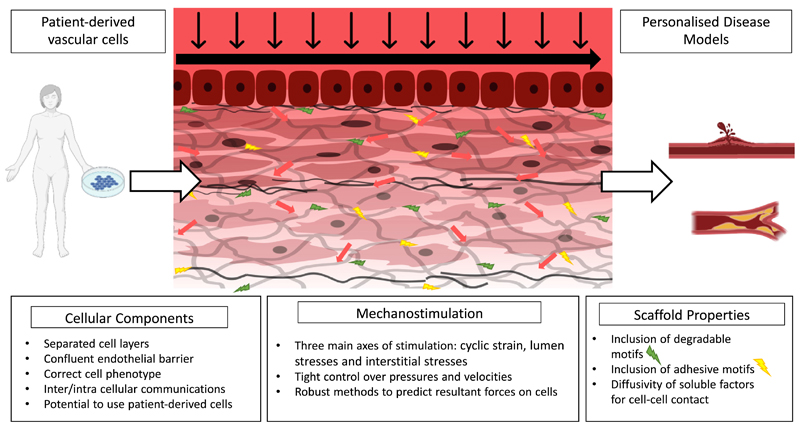
Schematic showing an ideal *in vitro* blood vessel model, which includes patient-derived vascular cells to study the effects of pathological stimuli for personalised disease modelling. Created with BioRender.com.

Building towards more representative *in vitro* experiments, the optimal way to simulate pathologies in arteries is likely to include all the pertinent stresses and strains with representative cell cultures in one setup (Fig. 7). Design of such a model is complex and requires careful consideration of how to incorporate these forces whilst retaining the ability to quantify them and control stimuli independently. Advances in the techniques outlined above may allow simultaneous 2D and 3D flow with different cell types to become increasingly feasible allowing for reductionist models which can incorporate all the major stimuli to vascular cells.

## 3 Future perspectives

Reductionist blood vessel models are proving to be useful tools in vascular disease modelling. These simplified setups may allow researchers to uncover underlying mechanisms that contribute to vessel pathologies, which can often be challenging to study in native tissue. However, by reducing the complexity of the cues in the *in vitro* environment, fidelity is limited, and potentially key stimuli may be overlooked. Therefore, conclusions should be critically appraised in the context of these limitations and verified using other approaches, if possible.

Undoubtedly, the mechanisms that underlie complex vascular diseases are multi-faceted and involve complex interactions between the factors highlighted in this review. Whilst studying cell cell interactions, cell-ECM interactions, or flow mechanotransduction independently may not be able to fully explain disease aetiology, by combining results obtained from *in vivo* studies with carefully constructed disease models, it may be possible to uncover factors that influence disease pathogenesis or progression. Indeed, elucidating the underlying mechanisms that drive vascular diseases may not only be important for understanding disease pathogenesis, but also key for identifying future therapeutic targets.

Not only can disease modelling potentially uncover underlying disease mechanisms, but the high controllability of the experimental conditions lends itself to use with techniques that may not be possible in tissue studies. Most notably, experimentally informed mathematical models are increasingly being applied in the fields of tissue engineering and disease modelling [168–170]. The concept underpinning such models is often the construction of a digital twin, whereby mathematical models constructed using experimental data predict biological behaviours, building in complexity as more experimental results are considered. *In silico* models can then be used to generate and test mechanistic hypotheses, study sensitivity to uncontrolled factors, and eventually produce *in silico* data that informs experimental decisions. Conversely, empirical data is used to parameterise and validate or extend the *in silico* models. A robust relationship can then be established between stimuli and cell behaviour through mathematical relations, and this can prove an extremely valuable tool for testing hypotheses around the mechanisms which underpin specific pathological behaviours. This combined approach, spanning *in vitro* experiments and mathematical models therefore not only serves to quantifiably characterise cell behaviours, but by building models capable of making validated predictions, can be used to reduce the number of experimental conditions required, ultimately expediting and reducing waste in *in vitro* work [168].

Furthermore, and particularly in the case of fluid forces, neither tangential nor interstitial shear can be experimentally measured. Therefore, quantification of mechanical forces is reliant on *in silico* work. However, creating robust mathematical models is reliant on reproducible, tightly controlled and characterisable setups. Given the need to work in 3D configurations, biomaterials are central to these models. The material’s ability to facilitate cell-matrix interactions is one of the limiting factors in creating viable 3D cultures and so creating bioactive synthetic hydrogels may be key.

Vessel models for studying disease progression may also allow the use of vascular cells differentiated from patient-derived induced pluripotent stem cells (iPSCs). Advances in cell differentiation techniques and organoid derivation and culture have led to an increase in the use of patient-specific cells for disease modelling, particularly in gastrointestinal, cardiac, neural, hepatic and renal disease [5, 141, 171–174]. Indeed, use of human iPSC to derive ECs for use in microvasculature have also emerged for use in organ-on-a-chip models [142, 175–178].

Patient-specific models offer obvious advantages in disease modelling, considering the role of genetics in cellular response to stimuli. Although some work has been made with use of iPSCs in cardiovascular applications, there is a paucity of work looking specifically at vessel disease [52, 179]. We suggest that advances in this research area may lead to an understanding of pathological stimuli in large vessel diseases specific to patient genetics and make strides towards personalised disease modelling.

## 4 Conclusion

This paper highlights the main techniques used to create *in vitro* blood vessels for disease modelling and their limitations. As of yet, no one model exists that takes into account all the pertinent factors present in vascular disease. Looking forward, there is a need to develop more comprehensive models combining the best advances in biomaterials, fluidic devices, computational models and stem cell research. Reductionist *in vitro* models using iPSC-derived patient cells could serve to advance personalised medicine and develop therapeutic interventions.

## Figures and Tables

**Table 1:** Table summarising the factors included and missing in some of the example blood vessel models described, highlighting that no one model includes all the factors necessary to comprehensively model vessel pathology.

Brief Description	Example Reference	Co-culture of VSMCs and ECs	Intercellular communication	3D culture of VSMCs	Cyclic Strain	Luminal shear	Interstitial shear through 3D matrix
Pulsatile flow passed over a layer of endothelial cells cultured on a stretchable membrane	[158]				x	x	
Monolayer of endothelial cells cultured on top of a layer of vascular smooth muscle cells separated by a layer of medial adhesion protein in a parallel plate biorector	[160]	x	x			x	
Monolayer of endothelial cells cultured on top of a layer of vascular smooth muscle cells separated by a porous membrane with flow passed over the cell cultures either side of the membrane	[1]	x	x		x	x	
Tubular scaffold seeded with vascular smooth muscle cells centered in a glass tube, pressurised from the inside to generate circumferential stretch, and perfused through the channel between the scaffold and glass wall to generate wall shear stress	[165]			x	x	x	
Flow driven through a 3D culture of cells embedded in a collagen matrix	[32,33]			x			x
